# Associations of GCLM, gclc and GSTP1 gene polymorphisms and antituberculosis drugs-induced hepatitis

**DOI:** 10.1186/1939-4551-8-S1-A151

**Published:** 2015-04-08

**Authors:** Sang-Heon Kim, Yong Eun Kwon, Sang-Hoon Kim, Jang Won Sohn, Ho Joo Yoon, Dong Ho Shin, Sung Soo Park, Jae Hyung Lee, Byoung-Hoon Lee, Youn-Seup Kim, Jae-Seuk Park, Young-Koo Jee

**Affiliations:** 1 Hanyang University College of Medicine, South Korea; 2 Chosun University College of Medicine, South Korea; 3 Eulji University School of Medicine, South Korea; 4 Hanyang University Hospital, South Korea; 5 Dankook University College of Medicine, South Korea

## Background

Antituberculosis drugs (ATD) is the most common cause of drug-induce liver injury in many countries. While the mechanism of ATD-induced hepatitis is poorly understood, oxidative stress is suggested to be involved in the development of liver injury to drug metabolites. In this regards, we explored the possible associations between glutathione related enzymes (*GCLM*, *GCLC* and *GSTP1*) gene polymorphisms and ATD-induced hepatitis.

## Methods

Through regular monitoring of liver function test during the treatment of tuberculosis, 84 patients with ATD-induced hepatitis and 237 ATD-tolerant controls were enrolled. Genotype were assessed in 3 single nucleotide polymorphisms in *GCLM* (rs41303970, -590T>C), *GCLC* (rs17883901, -594T>C) and *GSTP1* (rs1695, I105V) and compared between case and control groups.

## Results

No significant difference was found in genotype frequencies of rs41303970, rs17883901 and rs1695 between patients with ATD-induced hepatitis and ATD-tolerant controls in three statistical models (dominant, recessive and codominant model). In addition, the minor allele frequency were not different between case and control group in three polymorphism sites.

## Conclusions

There was no significant association between *GCLM*, *GCLC* and *GSTP1* gene polymorphisms and ATD-induced hepatitis. These findings suggest that genetic variants of GCLM, GCLC and GSTP1 do not increase the risk of ATD-induced hepatitis.

